# Supplementary material for: The effects of exercise training on circulating adhesion molecules in adults: A systematic review and meta-analysis
Source: PLoS One. 2023 Oct 13;18(10):e0292734. doi: 10.1371/journal.pone.0292734 (PMC10575525; doi:10.1371/journal.pone.0292734)
Supplement: S1 Table — (DOCX) [file pone.0292734.s002.docx]

**Supplementary Table 1. Search strategy**

| Databases | Search strategy | Limits | Results |
| --- | --- | --- | --- |
| PubMed | (("exercise"[All Fields] OR "physical activity"[All Fields] OR "exercise training"[All Fields] OR "aerobic training"[All Fields] OR "resistance training"[All Fields] OR "interval training"[All Fields] OR "High-intensity interval training"[All Fields] OR "concurrent training"[All Fields] OR "combined training"[All Fields]) AND ("inter cellular adhesion molecule*"[All Fields] OR "intercellular adhesion molecule*"[All Fields] OR "ICAM"[All Fields] OR "cell adhesion molecule"[All Fields] OR "CAM"[All Fields] OR "ICAM-1"[All Fields] OR "VCAM-1"[All Fields])) AND ((humans[Filter]) AND (english[Filter])) | Humans, English | 1239 |
| Scopus | ( TITLE-ABS-KEY ( "exercise"  OR  "training"  OR  "exercise training"  OR  "physical activity" )  AND  TITLE-ABS-KEY ( "caloric restriction"  OR  "weight loss"  OR  "diet"  OR  "dietary" )  AND  TITLE-ABS-KEY ( "high density lipoprotein cholesterol"  OR  "low density lipoprotein cholesterol"  OR  "Triglyceride"  OR  "total cholesterol"  OR  "cholesterol"  OR  "blood lipid"  OR  "blood fats"  OR  "lipid profile"  OR  "HDL"  OR  "LDL"  OR  "dyslipidemia"  OR  "hyperlipidemia"  OR  "hyperlipoproteinemia" )  AND  TITLE ABS-KEY ( "overweight"  OR  "obese"  OR  "obesity" )  AND  TITLE-ABS-KEY ( "randomized control trial"  OR  "randomized clinical trial"  OR  "randomized"  OR  "random*" ) ) | Article, English | 1696 |
| Web of science | (TS=("exercise" OR "physical activity" OR "exercise training" OR "aerobic training" OR "resistance training" OR "interval training" OR "High-intensity interval training" OR "concurrent training" OR "combined training")) AND TS=("inter cellular adhesion molecule*" OR "intercellular adhesion molecule*" OR "ICAM" OR "cell adhesion molecule" OR "CAM" OR "ICAM-1" OR "VCAM-1") and Article (Document Types) and English (Languages) | Article, English | 892 |
| Embase | ('exercise':ti,ab,kw OR 'physical activity':ti,ab,kw OR 'exercise training':ti,ab,kw OR 'aerobic training':ti,ab,kw OR 'resistance training':ti,ab,kw OR 'interval training':ti,ab,kw OR 'high-intensity interval training':ti,ab,kw OR 'concurrent training':ti,ab,kw OR 'combined training':ti,ab,kw) AND ('inter cellular adhesion molecule*':ti,ab,kw OR 'intercellular adhesion molecule*':ti,ab,kw OR 'icam':ti,ab,kw OR 'cell adhesion molecule':ti,ab,kw OR 'cam':ti,ab,kw OR 'icam-1':ti,ab,kw OR 'vcam-1':ti,ab,kw) | - | 1024 |
