# Supplementary material for: The effects of exercise training on circulating adhesion molecules in adults: A systematic review and meta-analysis
Source: PLoS One. 2023 Oct 13;18(10):e0292734. doi: 10.1371/journal.pone.0292734 (PMC10575525; doi:10.1371/journal.pone.0292734)
Supplement: S2 Table — (DOCX) [file pone.0292734.s003.docx]

Supplementary Table 2. Risk of bias assessment

| **Authors & Year** | Random sequence generation (selection bias) | Allocation concealment (selection bias) | Blinding of participants and personnel | Blinding of assessors | Incomplete outcome data | Selective outcome reporting | Other bias |
| --- | --- | --- | --- | --- | --- | --- | --- |
| Aksoy et al, 2015(38) | Low | Low | High | Unclear | Low | Low | Low |
| Andrade-Lima et al, 2021 (37) | Low | Low | High | Low | Low | Low | Low |
| Barone Gibbs et al, 2012 (36) | Unclear | Unclear | High | Unclear | High | Low | Low |
| Boeno et al, 2020 (26) | Low | Low | High | Low | Low | Low | Low |
| Brown et al, 2018 (35) | Unclear | Unclear | High | Low | Low | Low | Low |
| Byrkjeland et al, 2011 (34) | Low | Low | High | Unclear | Low | Unclear | Low |
| Byrkjeland et al, 2017 (33) | Low | Unclear | High | Unclear | Low | Low | Low |
| Castells-Sanchez et al, 2022 (19) | Low | Unclear | High | Low | Low | Low | Low |
| Connolly et al, 2016 (32) | Low | Low | High | Unclear | Low | Unclear | Low |
| Fernandes et al, 2011 (25) | Unclear | Unclear | High | Unclear | High | Low | Low |
| Hyun-Hun et al, 2019 (24) | Unclear | Unclear | High | Unclear | Unclear | Low | Low |
| Koh et al, 2018 (31) | Unclear | Unclear | High | Unclear | Unclear | Low | Low |
| Lim et al, 2015 (23) | Unclear | Unclear | High | Unclear | Low | Low | Low |
| Munk et al, 2011 (22) | Unclear | Unclear | High | Unclear | Low | Low | Low |
| Nikseresht et al, 2014 (18) | Unclear | Unclear | High | Unclear | Low | Unclear | Low |
| Olson et al, 2007 (30) | Unclear | Unclear | High | Unclear | Low | Low | Low |
| Ribeiro et al, 2012 (29) | Low | High | High | Low | Low | Low | Low |
| Rosety et al, 2016 (28) | Unclear | Unclear | High | Unclear | Unclear | Unclear | Low |
| Sjogren et al, 2010 (27) | Unclear | Unclear | High | Unclear | Unclear | Low | High |
| Soori et al, 2017 (17) | Unclear | Unclear | High | Unclear | Unclear | Low | Low |
| Timon et al, 2021 (21) | Unclear | Unclear | High | Unclear | Low | Low | Low |
| Vasic et al, 2019 (16) | Low | Low | High | Unclear | Low | Low | Low |
| Woudberg et al, 2018 (20) | Unclear | Unclear | High | Unclear | High | Low | Low |
