# Supplementary material for: The effects of exercise training on circulating adhesion molecules in adults: A systematic review and meta-analysis
Source: PLoS One. 2023 Oct 13;18(10):e0292734. doi: 10.1371/journal.pone.0292734 (PMC10575525; doi:10.1371/journal.pone.0292734)
Supplement: S3 Table — (DOCX) [file pone.0292734.s004.docx]

Supplementary Table 3. GRADE analysis of the overall quality of the evidence

**question.authors:** Mousa Khalafi, Michael E Symonds, Mohammad Hossein Sakhaei, Faeghe Ghasemi

**question.question:** Question.templates.IN_POP_sof

**question.setting:** Adults

**question.bibliography:**

| **outcome.certainty_assessment** | | | | | | | **outcome.no_of_patients** | | **outcome.effect** | | **outcome.certainty** | **outcome.importance** |
| --- | --- | --- | --- | --- | --- | --- | --- | --- | --- | --- | --- | --- |
| **outcome.no_of_studies** | **outcome.study_design** | **outcome.risk_of_bias** | **outcome.inconsistency** | **outcome.indirectness** | **outcome.imprecision** | **outcome.other_considerations** | **Exercise training** | **Control** | **outcome.relative (95% CI)** | **outcome.absolute (95% CI)** |  |  |
| **soluble intercellular adhesion molecule-1 (outcome.follow_up: outcome.range 2 outcome.weeks outcome.to 12 outcome.months; outcome.assessed_with: Elisa Kit)** | | | | | | | | | | | | |
| 16 | outcome.randomised_trials | outcome.serious^a^ | outcome.not_serious^b^ | outcome.not_serious^c^ | outcome.not_serious | outcome.publication_bias_strongly_suspected outcome.strong_association outcome.reduced_for_rr_long^d^ | 499 | 390 | - | SMD **0.33 SD outcome.lower** (0.56 outcome.lower outcome.to 0.11 outcome.lower) | ⨁⨁⨁⨁ Outcome.qualities.high | IMPORTANT |
| **soluble vascular cell adhesion molecule-1 (outcome.follow_up: outcome.range 2 outcome.weeks outcome.to 12 outcome.months; outcome.assessed_with: Elisa Kit)** | | | | | | | | | | | | |
| 19 | outcome.randomised_trials | outcome.serious^a^ | outcome.not_serious^e^ | outcome.serious^f^ | outcome.serious^g^ | outcome.publication_bias_strongly_suspected outcome.strong_association outcome.reduced_for_rr_long^d^ | 499 | 441 | - | SMD **0.12 SD outcome.lower** (0.29 outcome.lower outcome.to 0.05 outcome.higher) | ⨁⨁◯◯ Outcome.qualities.low | IMPORTANT |

outcome.printout.ci_only; outcome.printout.smd_only

#### footnotes.footnotes

a. Downgraded one level due to serious Risk of bias.

b. The I2 value was ≥50%, however, the higher heterogeneity was explained in the subgroup analyses.

c. Some studies were done on elderly adults and those with chronic disease; however, we performed subgroup analyses based on age and health status of participants

d. Both visual interpretation of funnel plots and Egger’s test results suggested publication bias.

e. The I2 value was < 50%.

f. Some studies were done on elderly adults and those with chronic diseases.

g. Failed to meet significant effect (CI includes SMD of ‘0’)
