# Supplementary material for: The effects of exercise training on circulating adhesion molecules in adults: A systematic review and meta-analysis
Source: PLoS One. 2023 Oct 13;18(10):e0292734. doi: 10.1371/journal.pone.0292734 (PMC10575525; doi:10.1371/journal.pone.0292734)
Supplement: Attachment — Submitted filename: Comments from the Editors and Reviewers post MES (1).docx [file pone.0292734.s005.docx]

Dear **Prof. Emily Chenette**,

We are grateful to you for giving this opportunity to revise our manuscript. We sincerely appreciate Reviewers’ efforts for their careful evaluation and valuable comments. Besides, your comments are very helpful to improve the quality of our manuscript.

According to the comments and directions, we have carefully revised our manuscript, and provide our point-by-point response to each comment. All the corrections in the revised manuscript were marked in ‘red’ for smooth follow-up. We believe that the changes we have done in our manuscript have improved the overall quality.

Sincerely yours,

Prof Michael E. Symonds

Centre for Perinatal Research, Academic Unit of Population and Lifespan Sciences,

School of Medicine, University of Nottingham, Nottingham NG7 2UH, United Kingdom.

Email: [michael.symonds@nottingham.ac.uk](mailto:michael.symonds@nottingham.ac.uk)

Review Comments to the Author

Reviewer #1:

**Comment**: #1 - Abstract - Methods - Language restrictions and the last search date should be stated.

**Response:** Amended as requested.

**Comment**: #2 - Abstract - Results/Conclusion - The authors should provide a general interpretation of the study findings and a brief summary of the limitations and significant implications of the evidence included in the review.

**Response:** Amended as requested.

**Comment**: #3 - Abstract - The protocol registered name, registration number, and where it can be accessed (e.g., Web address) should be provided in the Abstract.

**Response:** Amended as requested.

**Comment**: #4 - Methods - Last search date should be stated.

**Response:** Amended as requested.

**Comment**: #5 - Methods - Why did the authors not include other databases like Embase and CENTRAL? EMBASE used to be a relevant database for scoping/systematic reviews.

**Response:** Amended as requested. An electronic search was conducted in PubMed, Scopus, Web of Science and Embase databases and search were updated until 31 August 2023. Please see **Method- Search strategy** section**.**

**Comment**: #6 - Methods - Did the authors not consider a gray literature search, such as Google Scholar, relevant to this study question?

**Response:** The references list of selected studies and Google Scholar were manually searched to ensure that all relevant studies were included in the meta-analysis. Please see **Method- Search strategy** section**.**

**Comment**: #7 - Methods - Why did the authors include only articles in English?

**Response**: Limiting systematic reviews to English-only is common in systematic reviews and articles published in non-English languages require translation, which may often be accompanied by errors.

**Comment**: #8 - Methods - The authors should consider showing the complete search strategies for all databases, including any filters and limits used, in the Supplementary Material.

**Response:** Amended as requested. Please see supplementary table 1.

**Comment**: #9 - Methods - Were reference lists examined? If yes, the authors should specify the types of references examined.

**Response:** The references list of all selected studies and Google Scholar were manually searched to ensure that all relevant studies were included in the meta-analysis. Please see **Method- Search strategy section.**

#10 - Methods - What were the exclusion criteria? The inclusion and exclusion criteria should be explained in more detail using the study characteristics (e.g., participants, setting, target condition(s), and study design) and report characteristics (e.g., years considered, language, and publication status), preferentially showing the components of the PICO framework or one of its variant. A table summarizing the inclusion and exclusion criteria should be present.

**Response**: Added as requested. Please see Table 1.

#11 - Methods - Were unpublished manuscripts and conference abstracts eligible for inclusion?

**Response**: Unpublished manuscripts and conference abstracts were excluded.

#12 - Methods - The study selection process should describe in more detail in the text: duplicates, title, and abstract screening, and full-text articles assessment for relevance. Did the authors use a reference management software package or a screening and data extraction tool to study selection?

**Response**: Added as requested. Please see **Method- Eligibility Criteria and study selection**.

#13 - Methods - All independent variables and outcomes should be explicitly stated.

**Response**: Added as requested. Please see **Method- Eligibility Criteria and study selection**.

#14 - Methods - The authors used the Physiotherapy Evidence Database (PEDro) tool to assess the risk of bias of the included studies in the review. Did the authors consider adding an assessment to evaluate the quality of the evidence in the review, such as the GRADE framework?

**Response**: Added as requested.

Please see **Method- Quality assessment and sensitivity analyses selection**, **Results**- **Quality assessment and sensitivity of analyses section and supplementary Table 3.**

#15 - Discussion - Thea authors should discuss limitations at the study and outcome level (e.g., risk of bias) and the review level (e.g., incomplete retrieval of identified research, reporting bias).

**Response**: Added as requested. Please see **Discussion – Limitation section.**

#16 - Conclusion - The conclusion is too short. The critical implications of the evidence included in the review should be stated.

**Response**: Added as requested. Please see **Discussion – Limitation section.**

Reviewer #2:

Comment: The manuscript provides a solid basis for supporting the authors' findings, but an updated search is recommended. To evaluate the results, it is necessary to classify the quality of evidence using the GRADE method. It is necessary to ensure clear and legible resolutions for all figures and tables to avoid ambiguity.

**Response**: Amended as requested. An electronic search was conducted in PubMed, Scopus, Web of Science and Embase databases and search were updated until 31 August 2023. Please see **Method- Search strategy** section**.**

For GRADE, Please see **Method- Quality assessment and sensitivity analyses selection**, **Results**- **Quality assessment and sensitivity of analyses section and supplementary Table 3, and Figures.**
